# Determining the accuracy of preoperative total hip replacement 2D templating using the mediCAD^®^ software

**DOI:** 10.1186/s13018-022-03086-5

**Published:** 2022-04-10

**Authors:** Seyed Peyman Mirghaderi, Sadula Sharifpour, Alireza Moharrami, Negar Ahmadi, Rangarirai Makuku, Maryam Salimi, Seyed Mohammad Javad Mortazavi

**Affiliations:** 1 Joint Reconstruction Research Center (JRRC), Imam Khomeini Hospital, Tehran University of Medical Sciences, End of Keshavarz Blvd, 1419733141 Tehran, Iran; 2 Students’ Scientific Research Center (SSRC), Tehran University of Medical Sciences, Tehran, Iran

**Keywords:** Arthroplasty, Hip, Total hip replacement, mediCAD^®^, Templating, Preoperative planning

## Abstract

**Background:**

Templating is a preoperative planning procedure that improves the efficiency of the surgical process and reduces postoperative complications of total hip arthroplasty (THA) by improving the precision of prediction of prosthetic implant size. This study aimed to evaluate the accuracy of the preoperative cup and stem size digital 2D templating of THA with mediCAD^®^ software and find the factors that influence the accuracy, such as indication for surgery, patients’ demographics, implant brand, and the assessors’ grade of education.

**Methods:**

We retrospectively retrieved 420 patient template images of all patients who underwent THA between March 2018 and March 2021. Templating of all included images was processed using mediCAD^®^ software a day before surgery by a newcomer physician to hip arthroplasty course (PGY-2 orthopedic resident or hip surgery fellow). Preoperative templating cup and stem sizes were compared with the actual inserted implant sizes.

**Result:**

After excluding ineligible patients, this study included 391 patients, 193 (49.4%) males and 198 (50.6%) females with a mean age of 43.3 ± 14.9. The average cup sizes predicted before and after surgery were 52.12 ± 14.28 and 52.21 ± 15.05 respectively, and the mean delta cup size (before and after surgery) was 2.79 ± 2.94. The delta stem size before and after surgery has a mean value of 1.53 ± 1.49. The acetabular cup components, measured within ± 0, ± 1, and ± 2 sizes, were 28.9%, 63.9%, 83.1% accurate, respectively. The femoral stem design component measured within ± 0, ± 1, and ± 2 sizes were 27.2%, 61.0%, 78.6% accurate, respectively. Wagner Cone^®^ stem brand, DDH patients, and females showed significantly higher accuracy of stem size templating. Revision THA has the lowest accuracy in terms of cup size templating. The compression of accuracy rate between resident and fellow revealed no significant differences. Also, no significant difference was detected between the accuracy of templating performed in the first months with the second months of the arthroplasty course period.

**Conclusion:**

Our study showed that under mentioned condition, templating using mediCAD^®^ has acceptable accuracy in predicting the sizes of femoral and acetabular components in THA patients. Digital software like mediCAD^®^ remains favorable because of the short learning curve, user-friendly features, and low-cost maintenance, leading to level-up patient care and THA efficacy. Further studies are necessary for clarifying the role of the assessor’s experience and expertise in THA preoperative templating.

**Level of evidence:**

Level III (retrospective observational study).

## Introduction

Hip pathology management evolved from rudimentary joint excision and osteotomies to modern-day total hip arthroplasty (THA) [1, 2]. THA is now the gold standard procedure for orthopedic patients with extensive hip joint pathologies such as developmental dysplasia of the hip [3] and end-stage hip osteoarthritis [4]. Although the most common indication of THA is osteoarthritis, the procedure is also indicated for the management of displaced fracture of the femoral neck in younger patients, juvenile rheumatoid arthritis, ankylosing spondylitis, hip fractures, bone tumors (benign and malignant), arthritis that is associated with Paget’s disease, and unreduced traumatic dislocation of the hip joint [5].

One of the latest, common, and essential improvements to THA pre-planning procedures has been digital preoperative templating [1–4]. Templating is a preoperative planning procedure that improves the efficiency of the surgical process and reduces postoperative complications of THA. Preoperative templating improves the precision of predicting correct prosthetic implant size [5–7]. Moreover, preoperative planning is helpful to predict the implant position and alignment, restore the center of rotation and equalize limb length [8]. This will help avoid limb discrepancies, inadequate offset reconstructions, fractures, and implant failures due to instability [6]. For instance, selecting an undersized femoral component may lead to femoral neck notching or varus implant alignment [9]. Studies have uncovered additional advantages of templating, such as an increase in the success and survival rates of the procedure, decreasing surgical time, preventing bone loss, periprosthetic fractures, and early exclusion of unavailable or sub-standard implants [10].

Earlier, preoperative templating was done by drawing on transparencies of appropriately magnified implants provided by the prosthesis manufacturer [11]. This analog templating used preoperative hard-copy film-based radiographs and transparencies lined up in the desired orientation to identify the appropriate size of the implant [11]. The development of digital image acquisition techniques and digital image review ushered a new era of preoperative planning backed by radiography and software replacing the previous practice [12]. This advancement has improved templating accuracy to predict the implant size, position, alignment, and restoration of the center of rotation and equalize limb length [6, 13, 14]. Digital planning is more reliable and accurate than analog [14]. It has been observed that preoperative templating has increased the success of planning the size of the instrument in THA to almost 98% [5].

Currently, there are three different options for templating: acetate on digital images, 2D templating on digital images, and digital 3D templating [15, 16]. Several commercialized software that allows digital planning is available; examples include King Mark [5], mediCAD-system [17] MATLAB software from MathWorks [18], Merge Healthcare [11], Apple’s Keynote presentation software [19], TraumaCad™ system [20], EndoMap software [21] and EOS imaging [22]. This software differs in time efficiency, costs, and accuracy, which are important factors for healthcare providers’ prioritization for acquisition. Numerous companies have developed software to computerize the process since 1996, and mediCAD^®^ was the first commercially available, specializing mainly in the German market. Until 2003, the market only truly developed when OrthoView^®^, from the UK, was founded by Albany Ventures, and TraumaCad^®^, from Israel. Based on the properties of the most popular programs (MediCad, TraumaCad, and OrthoView), all of them are capable of measuring most orthopedic parameters. All these software facilitate geometric planning, which can be accomplished using surface models [23–25].

A progressive improvement in the accuracy of THA prediction has resulted from a shift from 2 to 3D preoperative planning. The current 3D planning software operation procedures are complicated, subjective, and time-consuming. Consequently, artificial intelligence (AI) has found its way into orthopedic templating with the advancement of technology. PeekMed^®^ implemented an AI-based system in 2015, which speeds up and automates several time-consuming and cumbersome steps by automatically segmenting the bones, finding landmarks and planning the surgical procedure, using the best surgical practices, and quickly choosing the correct correction and implant. Another applicable software based on AI is the AI HIP; using AI technology, prosthesis designs can automatically detect acetabular and femoral morphology and match prosthesis sizes [26, 27]. As accurate as 3D software, it takes significantly less time.

For the cup, 2D templating methods were able to determine the implant size within a range of 25% and 85.7%, while for the stem, it was within a range of 32% and 49.15%. In terms of predicting the accurate size of the implant within one size, these values ranged from 45 to 89.3% for the cup and from 60.7 to 83.6% for the stem. The accuracy is significantly higher when using 3D templating methods [16]. This study aimed to evaluate the accuracy of preoperative templating of THA using the mediCAD^®^ software. We aim to indicate the accuracy of stem and cup size pre-planning within ± 0, ± 1, and ± 2 size and find the factors that influence the accuracy.

## Material and methods

This study was approved by the Ethics Committee of Tehran University of Medical Sciences. In this retrospective cross-sectional investigation, we retrieved 420 consecutive digital radiographs from the Picture Archiving Communication System (PACS) of all patients who underwent THA between March 2018 and March 2021 in our orthopedics department. This study was undertaken at the Imam Khomeini Hospital Complex (IKHC), the largest referral and teaching hospital in Iran. The hospital’s orthopedic center has performed preoperative planning and templating for THA patients for over a decade. However, up to now, no investigation on the accuracy of the procedure has been conducted.

**Fig. 1:**
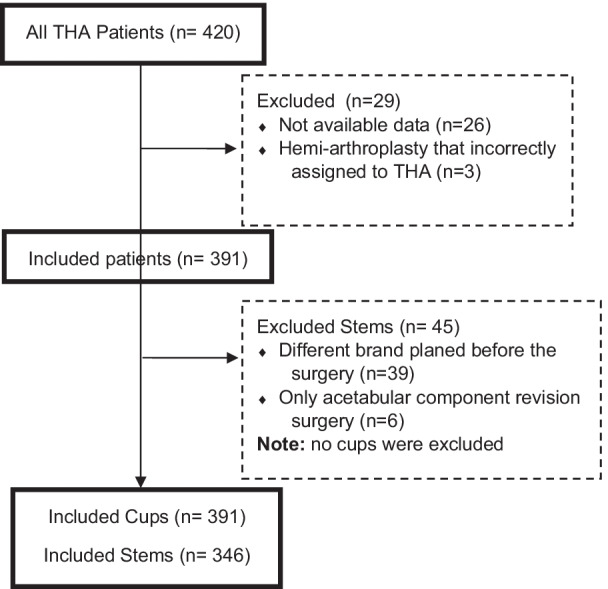
Patient enrolment flow diagram

After including all THA patients having templated before surgery in that period, by searching the archive database, 29 patients were excluded for lack of data (*n* = 26) and inappropriate assignment to THA (*n* = 3), and remained 391 (Fig. 1). Patients with severely impaired hip anatomy (e.g., fused hip, proximal femoral deformity, untreated spinal deformity, etc.) were not initially included as they were not templated before THA. They have not templated before THA due to the complexity of preoperative templating for a newly trained resident or fellow. All patients underwent cementless THA under the same condition and all through a direct anterior approach by a single surgeon (SMJ.M).

**Fig. 2:**
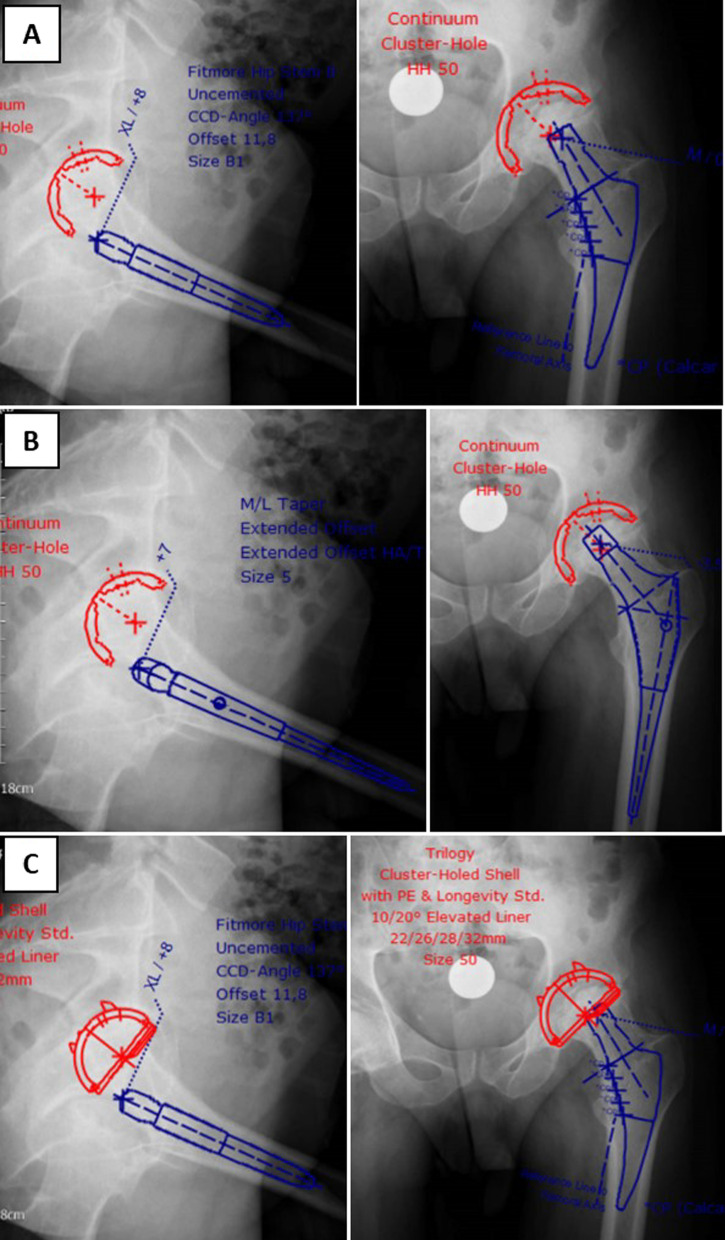
Sample templated using mediCAD^®^ software. Anteroposterior (right) and lateral (left) views. **A** Continuum^®^ cup and Fitmore^®^ stem. **B** Continuum^®^ cup and M/L taper stem. **C** Trilogy^®^ cup and Fitmore^®^ templating

**Fig. 3:**
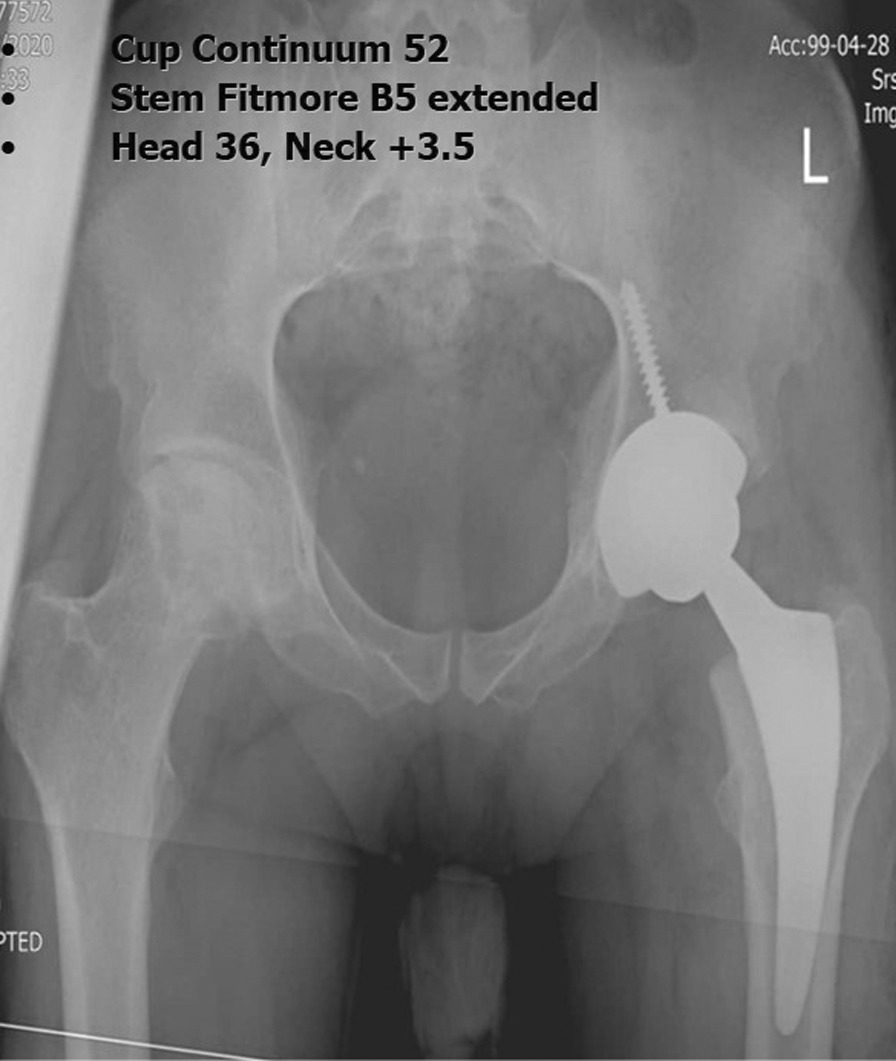
Postoperative radiograph and implant details

Preoperative planning for all cases was carried out using the mediCAD^®^ software Version 5.98 (Hectec, Niederviehbach, Germany). This software allowed scaling the image with a marker of known size and applying it to the digital template to predict the specifications of planned endoprosthesis elements. mediCAD^®^ also helped choose the implants based on an analysis of hip joint-specific anatomy and bone stock conditions. Each patient underwent preoperative AP X-rays of the pelvis according to the same protocol in the same radiology department. Each patient was lying supine, both legs in an internal rotation of 15°. The distance between the tube and film was standardised at 1.15 m. The beam was placed in the center of the symphysis pubis. Marker ball technique was used to standardize the X-rays (Fig. 1), all taped to the skin in the same position. All preoperative radiographs were templated a day before surgery by a newcomer physician to a hip arthroplasty course (PGY-2 orthopedic resident or new hip and pelvis surgery fellow, *n* < 500 THA). They began to template the THA first time in the arthroplasty course of their residency/fellowship program at IKHC. Each resident stays at the arthroplasty ward for 2 months and practices templating with the software. On the other hand, the newcomer fellow practiced templating with the software for 6 months at the beginning of their fellowship program. Pre- and postoperative radiographs (AP and lateral) were obtained with a standard source-to-object of 2.5 cm calibration metal ball. For each patient, they were templating performed for more than one prosthesis implant type because due to limitations or changing surgeon’s decision, the implant brand changed on the day of the surgery (Figs. 2, 3). Cup brands used in templating included Continuum^®^ (Zimmer Biomet, USA), PINNACLE^®^ (DePuy, USA), Trident^®^ (Stryker, USA), Trilogy^®^ (Zimmer Biomet, USA), Trilogy^®^ IT(Zimmer Biomet, USA). Stem types include Fitmore^®^ (Zimmer Biomet, USA), M/L taper (Zimmer Biomet, USA), Wagner Cone^®^ (Zimmer Biomet, USA), Accolade^®^ (Stryker, USA), CORAIL^®^ (DePuy, USA), Müller™ (Zimmer Biomet, USA), and Wagner SL Revision^®^ (Zimmer Biomet, USA). Implant’s brand that used in surgery was considered for calculating the accuracy. During the intraoperative process, the surgeon selected the implant size based on the patient’s anatomy, joint stability, and leg length determined from the preoperative X-rays. For stem accuracy calculating, the patient was excluded if none of the preoperative templating brands was used on the day of the surgery. A 1 mm difference exists between the stem’s sizes and a 2 mm difference for the cup.

### Statistical analysis

Preoperative templating measurements and actual sizes used during the operation were first recorded in an excel sheet and later transferred to IBM SPSS software version 22 for computations and analysis. Descriptive statistics of continuous variables were expressed as mean (SD), while categorical data were expressed as numbers and percentages. We analyzed data for accuracy using the Chi-square test, post-hoc analysis with Bonferroni correction, and independent sample *T* test. Statistical significance was tested using a *P* value of 0.05 were necessary.

## Results

### Patient demography

Our investigation consisted of 391 patients, 193 (49.4%) males and 198 (50.6%) females, with an average age of 43.3 ± 14.9 and a Body Mass Index (BMI) of 24.6 ± 4.4. The most common indication for THA surgery at our center during the period under study was avascular necrosis (AVN) with 100 (25.6%) patients, followed by developmental dysplasia of the hip (DDH) 198 (50.6%), degenerative joint disease (DJD) 37 (9.5%), Fracture 19 (4.9%), Revision 24 (6.1%), and Others 13 (3.3%).

Excluding 45 patients for calculating stem size, preop-planning accuracy resulted in 346 patients: 39 excluded for different brands planned before the surgery and six excluded for only acetabular component revision surgery. We used eight brands of the femoral component prosthesis. Fitmore^®^ was the most used brand with 120 (27.8%) stems, followed by Fitmore^®^ extended 71 (16.5%), M/L taper 51 (11.8%), Wagner Cone^®^ 48 (11.1%) Accolade^®^11 (2.6%), CORAIL^®^ 61 (14.2%), Müller™ 2 (0.5%), Wagner SL Revision^®^ 21 (4.9%), and six unknown brand of stem due to lack of data in the archive. We enrolled 391 acetabular components from 5 main different cup brands; Continuum^®^ 226 (52.4%), Pinnacle® 63 (14.6%), Trident^®^ 11 (2.6%), Trilogy^®^ 81 (18.8%), and Trilogy^®^ IT 7 (1.6%). Other minor types of 3 (0.7%) included Cage *N* = 1, shell *N* = 1, and Dual mobility *N* = 1 cups.

### Prediction of the femoral component (stem) size

The accuracy of the femoral stem design component measured within ± 0, ± 1, and ± 2 sizes increased from 27.2%, 61.0%, and 78.6%, respectively (Table 1). The delta stem size before and after surgery has a mean value of 1.53 ± 1.49.

**Table 1:** Cup and stem size templating accuracy in different conditions

	Patients	Cup accuracy (*N* = 391)			Stem accuracy (*N* = 346)		
		± 0	± 1	± 2	± 0	± 1	± 2
Total	391 (100%)	28.9%	63.9%	83.1%	27.2%	61.0%	78.6%
*Nominal variables*							
Year							
2018	67 (17.1%)	26.9%	61.2%	82.1%	32.7%	75.0%	88.5%
2019	122 (31.2%)	33.6%	66.4%	86.9%	28.2%	60.0%	82.7%
2020	139 (35.5%)	26.6%	63.3%	80.6%	23.8%	54.8%	71.4%
2021	63 (16.1%)	27%	63.5%	82.5%	27.6%	63.8%	77.6%
*P* value*		0.590	0.904	0.585	0.663	0.087	0.046 (post-hoc analysis revealed no significance)
Gender							
Male	193 (49.4%)	30.1%	65.8%	85.0%	20.8%	53.8%	76.3%
Female	198 (50.6%)	27.8%	62.1%	81.3%	**33.5%**	**68.2%**	80.9%
*P* value*		0.620	0.448	0.334	**0.008**	**0.006**	0.294
Indication for surgery							
AVN	100 (25.6%)	24.0%	69.0%	83.0%	18.8%	49.0%	70.8%
DDH	198 (50.6%)	31.8%	65.7%	86.9%	32.2%	70.7%***	84.5%
DJD	37 (9.5%)	27.0%	64.9%	81.1%	28.6%	60.7%	75.0%
Fracture	19 (4.9%)	21.1%	47.4%	84.2%	23.5%	58.8%	70.6%
Revision	24 (6.1%)	41.7%	54.2%	62.5%***	22.2%	44.4%	72.2%
Other (acetabular fracture *N* = 1, hip fusion *N* = 2, hemophilia DJD *N* = 1, perthes *N* = 6, septic arthritis *N* = 3)	13 (3.3%)	15.4%	38.5%	69.2%	30.8%	46.2%	84.6%
*P* value*		0.339	0.141	**0.048**	0.297	**0.007**	0.126
Stem brand (*N* = 391, 346 was considered for accuracy estimation)							
Fitmore^®^	120 (27.8%)	22.5%	65.0%	84.2%	21.2%	45.2%***	71.2%
Fitmore^®^ extended	71 (16.5%)	26.8%	62.0%	84.5%	25.7%	60.0%	77.1%
M/L taper	51 (11.8%)	29.4%	62.7%	86.3%	34.8%	67.4%	82.6%
Wagner Cone^®^	48 (11.1%)	**52.1**%***	72.9%	87.5%	41.7%	81.3%***	91.7%
Accolade^®^ ****	11 (2.6%)	9.1%	36.4%	72.7%	–	–	–
CORAIL^®^	61 (14.2%)	24.6%	67.2%	82.0%	23.7%	72.9%	83.1%
Müller™ ****	2 (0.5%)	0.0%	100.0%	100.0%	–	–	–
Wagner SL revision^®^	21 (4.9%)	38.1%	42.9%	61.9%	21.1%	47.4%	68.4%
Unknown brand (lack of data)	6 (1.5%)	–	–	–	–	–	–
*P* value*		**0.006**	0.153	0.321	0.104	**0.001 < **	0.058
Cup brand							
Continuum^®^	226 (52.4%)	31.4%	60.2%	82.3%	30.1%	61.2%	78.5%
PINNACLE^®^	63 (14.6%)	25.4%	66.7%	81.0%	23.0%	72.1%	82.0%
Trident^®^	11 (2.6%)	9.1%	36.4%	72.7%	–	–	–
Trilogy^®^	81 (18.8%)	28.4%	76.5%	88.9%	25.4%	49.3%	77.6%
Trilogy^®^ IT	7 (1.6%)	14.3%	57.1%	85.7%	0.0%	57.1%	57.1%
Other (cage *N* = 1, shell *N* = 1, Dual mobility *N* = 1)	3 (0.7%)	33.3%	66.7%	66.7%	0.0%	100.0%	100.0%
*P* value*		0.558	0.052	0.593	0.292	0.078	0.572
*Continuous variables (mean of variable in the accurate group versus inaccurate group)*							
Age (mean ± SD)		44.7** ± **13.8 versus 42.8** ± **15.3	43.9** ± **14.1 versus 42.3** ± **16.3	43.5** ± **14.8 versus 42.2** ± **15.5	41.2** ± **14.2 versus 43.4** ± **15.0	42.2** ± **14.6 versus 43.8** ± **15.1	42.5** ± **14.7 versus 44.1** ± **15.2
*P* value**		0.252	0.332	0.508	0.216	0.313	0.410
BMI (Kg m^−2^, mean)		25.0** ± **4.0 versus 24.5** ± **4.5	24.4** ± **4.2 versus 25.0** ± **4.6	24.5** ± **4.4 versus 25.2** ± **4.3	24.5** ± **4.3 versus 24.6** ± **4.3	24.5** ± **3.9 versus 24.5** ± **4.6	24.4** ± **4.4 versus 24.9** ± **3.8
*P* value**		0.249	0.183	0.243	0.919	0.972	0.428
Cup diameter (mm, mean)		51.2** ± **4.5 versus 52.5** ± **4.1	51.9** ± **4.1 versus 52.5** ± **5.6	52.0** ± **4.2 versus 52.6** ± **4.7	51.1** ± **4.4 versus 52.5** ± **4.2	51.5** ± **4.4 versus 53.1** ± **3.9	51.8** ± **4.4 versus 53.2 ± 3.8
*P* value**		**0.007**	0.195	0.346	**0.008**	**0.001 > **	**0.012**

Bold values are statistically significant values

*P* value* = Chi-square test

*P* value** = independent sample *T* test

***Significant after post-hoc analysis with corrected Bonferroni *P* value

****Not included in the chi-square analysis due to low number of population

**Fig. 4:**
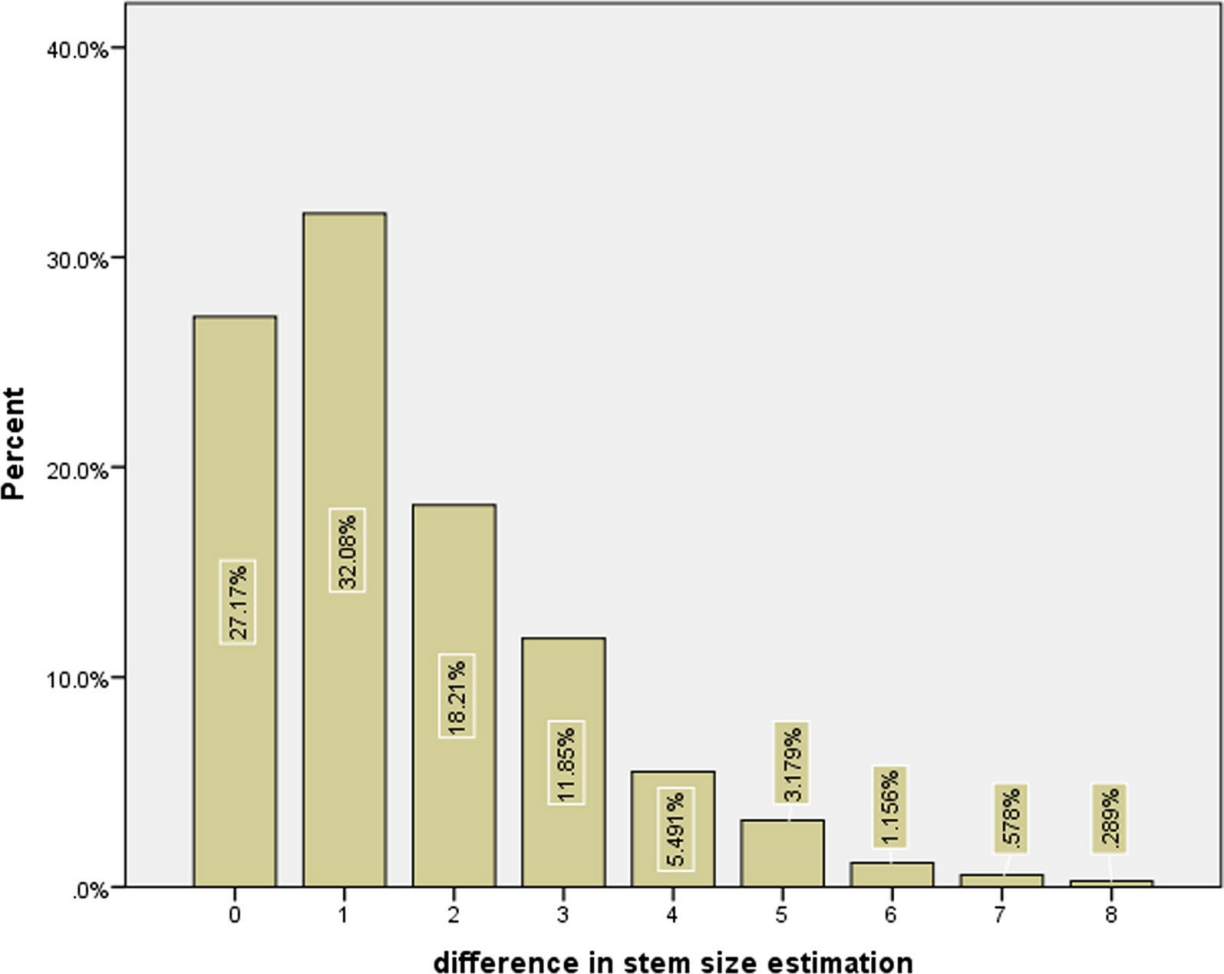
Bar graphs showing differences in stem size estimation and percent of patients with this difference

Brand groups showed the highest accuracy in their all sizes with Wagner Cone^®^(41.7%, 81.3%, and 91.7%) that only in ± 1 size accuracy was significant (81.3%, *P* < 0.001). Fitmore^®^ implant has significantly lowest ± 1 size accuracy with 45.2% (*P* < 0.001). Regarding surgery indication, DDH patients have significantly highest ± 1 size accuracy with 70.7% (*P* = 0.007). Females showed higher ± 1 and ± 0 accuracy compared to males (*P* < 0.05). Table 1 shows further information on the distribution of accuracies among other sizes. Figure 4 shows the percent of patients that exist in each interval difference between predicted and actual stem size. The accuracy for stem type was recorded as 90.0% (346 out of 385 stems have the same type of inserted stem that had been templated before the surgery).

### Prediction of the acetabular component (cup) size

**Fig. 5:**
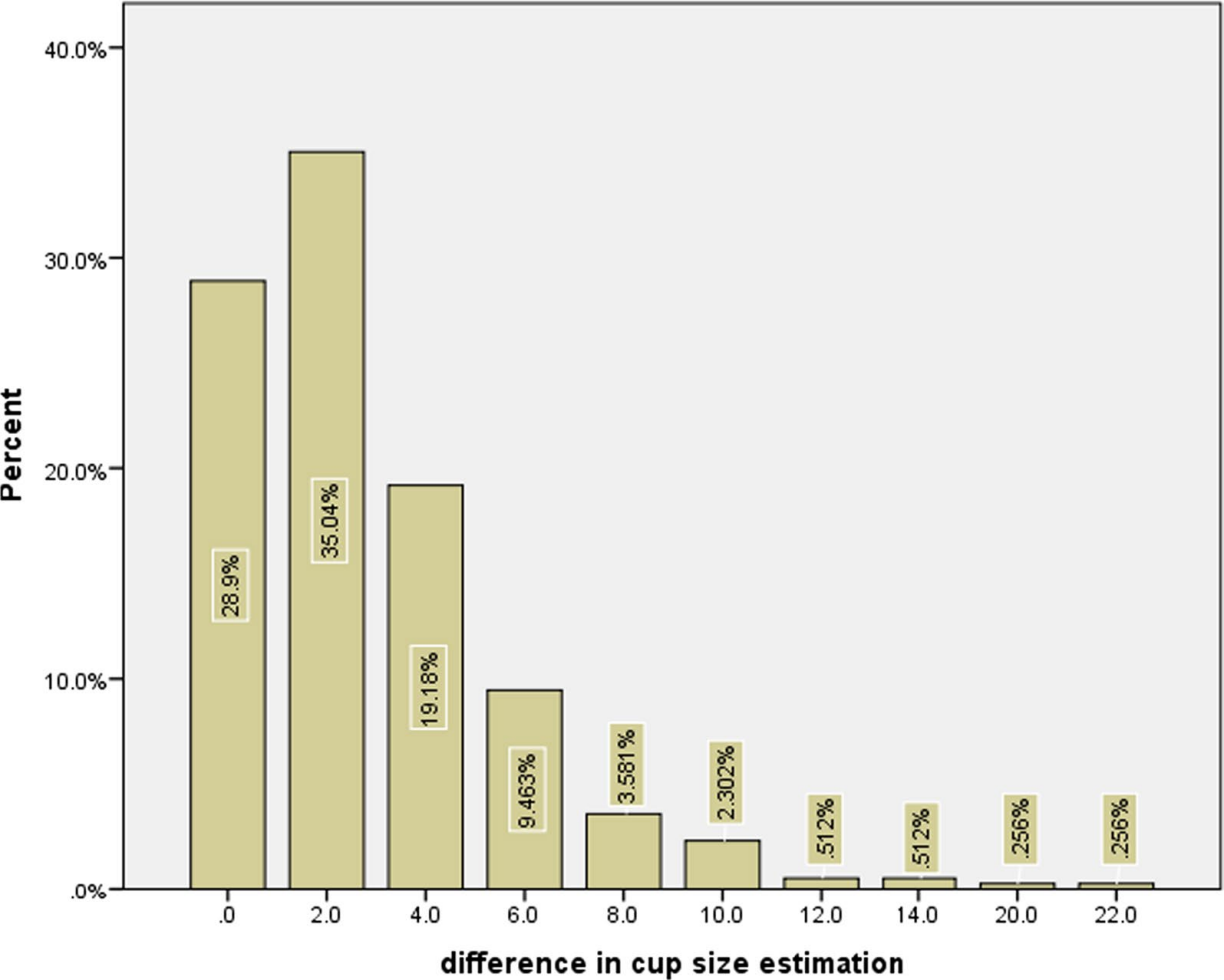
Bar graphs showing differences in cup size estimation and percent of patients with this difference

The average actual and predicted cup sizes used were 52.21 ± 5.05 and 52.12 ± 4.28 mm, respectively. The mean delta cup size (before and after surgery) was 2.79 ± 2.94. The accuracy of acetabular cup components ± 0, ± 1, and ± 2 sizes were 28.9%, 63.9%, 83.1%, respectively. The highest templating accuracy ± 1 rate of the acetabular component within brands was observed in Trilogy^®^ group with 76.5%, followed by PINNACLE^®^ 66.7%, and the least was Trident^®^ with 36.4% but statistically insignificant (*P* = 0.052). The ± 0 accurate group has a significantly smaller cup size than the non-accurate group (51.2 vs. 52.5, *P* = 0.007). Figure 5 shows the percent of patients that exist in each interval difference between predicted and actual cup size.

### Cup and stem size templating accuracy: resident versus fellow

The compression of accuracy rate between resident and fellow was represented in Table 2. No significant differences were observed among them in almost all accuracies (*P* > 0.05), except for cup ± 1 size accuracy that residents were higher (68.9% vs. 59.1%; *P* = 0.043). Also, no significant difference was detected between the accuracy of templating performed in the first months with the second months of the arthroplasty course period (*P* > 0.05).

**Table 2:** Cup and stem size templating accuracy; comparing resident and fellow

	Patients	Cup accuracy (*N* = 391)			Patients	Stem accuracy (*N* = 346)		
		± 0	± 1	± 2		± 0	± 1	± 2
Total	391 (100%)	28.9%	63.9%	83.1%	346 (100%)	27.2%	61.0%	78.6%
Physician								
Resident	193 (49.4%)	30.1%	68.9%	85.5%	163 (47.1%)	28.2%	57.7%	78.5%
Fellow	198 (50.6%)	27.8%	59.1%	80.8%	183 (52.9%)	26.2%	63.9%	78.7%
*P* value*		0.62	**0.043**	0.22		0.68	0.23	0.97
Month at arthroplasty course (first vs. second)	273 (100%)				238 (100%)			
First month	118 (43.2%)	26.3%	71.2%	89.0%	100 (42.0%)	31.0%	58.0%	76.0%
Second month	155 (56.8%)	33.5%	63.9%	82.6%	138 (58.0%)	25.4%	55.8%	79.0%
*P* value**		0.20	0.20	0.14		0.34	0.74	0.59
Fellow templating	198 (100%)				183 (100%)			
First 3-months	114 (57.6%)	30.7%	63.2%	85.1%	106 (57.9%)	24.5%	63.2%	79.2%
Second 3-months	84 (42.4%)	23.8%	53.6%	75.0%	77 (42.1%)	28.6%	64.9%	77.9%
*P* value*		0.29	0.18	0.08		0.54	0.81	0.83

Bold values are statistically significant values

*P* value* = Chi-square test

### Relationship between the cup and stem accuracy

Table 3 shows the association of stem and cup size prediction accuracy within ± 1 size. 41.3% of cases are templated accurately within one size for both components. No significant association was detected (*P* = 0.059).

**Table 3:** Cup and stem size templating accuracy within ± 1 size relationship

*P* = 0.059	Accuracy stem ± 1		Total
	No	Yes	
Accuracy cup ± 1			
No	57 (16.5%)	68 (19.7%)	125 (36.1%)
Yes	78 (22.5%)	143 (41.3%)	221 (63.9%)
Total	135 (39.0%)	211 (61.0%)	346 (100.0%)

*P* value* = Chi-square test

## Discussion

The introduction of digital preoperative templating in THA has improved clinical and technical outcomes of predicting actual prosthetic implant sizes [28]. This has resulted in several benefits, including fewer postoperative complications, less procedure time, and costs [10, 29]. Nowadays, many templating approaches and software are on the market, with varying accuracies. Although latest studies report three-dimensional CT-based software having better prediction accuracy than 2D digital templating, higher radiation exposure remains a critical demerit [16]. This study aimed at evaluating the accuracy of preoperative digital 2D templating of THA with mediCAD^®^ software and find the factors that influence the accuracy, including indication for surgery, patients’ demographics, implant brand, and the assessors’ grade of education.

Our investigation consisted of 391 patients, 193 (49.4%) males and 198 (50.6%) females. To our knowledge, we studied the largest sample of patients under assessing the accuracy of preoperative templating. This investigation found that using mediCAD^®^, the accuracy of the femoral stem design component measured within ± 0, ± 1, and ± 2 sizes were 27.2%, 61.0%, and 78.6%, respectively. The accuracy of acetabular cup components measured within ± 0, ± 1, and ± 2 sizes were 28.9%, 63.9%, 83.1%, respectively.

Our accuracy rates were low compared to those recorded in earlier investigations (Table 4). The reasons for low templating accuracy in our dataset may have been due to human error in the templating process, as reported by Wiese et al. [30]. Additionally, Carter et al., Bertram The et al., and Choi et al. have reported that surgeon experience has a significant role in the accuracy of predicting component size during templating [7, 14, 31]. In contrast, Efe et al. and Shin et al. found no difference in preoperative planning accuracy according to the planning surgeon’s level of training [32, 33]. Since amateur assessors were pre-planning at our center, the experience might have been an essential factor against accuracy. Additionally, there were no significant differences between the accuracy of PGY-2 residents and the newcomer's hip and pelvis fellows in almost all accuracies except for cup ± 1 size accuracy. Even in the cup, ± 1 size accuracy residents were superior. PGY-2 residents are supposed to have more experience with our center and also receive more assistance from the more senior residents so they may be more familiar with the software and templating. Fellows came from other centers that didn't do templating before THA or were far from templating for years. All in all, the results may suggest that expertise in orthopedics is not enough to improve accuracy in templating, and the role of expertise in using the software and templating is critical. On the other hand, there was not a significant improvement in accuracy when comparing the first month of using the software with the second month for assessor. This may stem from the fact that training for a month is not enough for accurately template the implant size before THA.

**Table 4:** Previous accuracy measurements reported from different studies and the current study

Study’s author	Sample size	Type templating	Cup size accuracy % (± 0/ ± 1/ ± 2)	Stem size accuracy % (± 0/ ± 1/ ± 2)	Citation
Current study	391	Digital	28.9/63.9/83.1	27.2/61.0/78.6	Current study
Wedemeyer et al.	40	Digital	40/77.5/92.5	37.5/95/100	[37]
Davila et al.	36	Digital	39/86/94	19/72/94	[21]
EL Steinberg et al.	73	Digital	50.7/89/100	–/87/96	[20]
Kristoffersson E et al.	50	Digital	42.9/80.4/	38.2/81.8/	[36]
Carter et al.	64	Digital	–	43.2/82.4/95.9	[7]
Eggli et al.	100	Scanned films-software	–/90/–	–/92/–	[40]
Dutka J et al.	348	Analog radiograms	85* (± 1)	77*(± 1)	[41]
		Digital	82(± 1)	72(± 1)	
Choi JK et al.	80	Digital	37.8/80.6/–	64.4/98.8/–	[31]
Gamble P et al.	117	Digital	38/80/–	35/85/–	[35]
		Onlay templating	20/60/–	40/85/–	
Efe T et al.	169	Digital	33.7/77.5/–	36/82.3/–	[32]
Bertram Theet al.	238	Digital (cemented)	72(± 1)	79(± 1)	[14]
		Digital (uncemented)	52(± 1)	66(± 1)	
Whiddon DR et al.	51	Digital	39/78/96	61/90/96	[34]
		Manual	67% (± 1)	82% (± 1)	
Shaarani SR et al.	100	Digital	38/80/98	36/75/98	[42]
Shin JK et al.	200	Digital	65/96.6/–	69.1/97.8/–	[33]

High BMI has been mentioned as a contributory factor to causing significant margins of error in digital planning; however, the average BMI investigated in this study is relatively lower than in earlier studies [34]. Although our (± 1) cup size accuracy (63.9%) was generally not high (Table 2), it was higher than EL Steinberg et al. with 50.7% [20]. Our average cup measurements before and after the surgery (52.12 and 52.21) were closely related to EL Steinberg et al.’s (51.5 ± 3.3 and 52 ± 2.9), respectively [20]. As all patient included in the study was uncemented THA, the other source of templating error could be the procedure’s cementless origin. It has been shown that cementless components templating has lower accuracy than cemented, possibly coming from the press-fit technique for the latter [35].

In the current study, DDH patients showed higher accuracy than other patients. Comparing to Kristofersson et al. study on DDH patients, the cup size accuracy (± 0: 31.8% vs 42.9%, ± 1: 65.7% vs 80.4%) is lower here, however stem size accuracy (± 0: 32.2% vs 38.2%, ± 1: 70.7% vs 81.8) was comparable. They found no association between the severity level of DDH and implanted and templated cup size difference [36]. Also, in line with them, the cup accuracy was higher than stem in this study. However, some other studies showed reverse results [31]. Disrupted femoral anatomy in DDH patients is a reason that stem accuracy is lower than a cup in DDH individuals [36].

The Wedemeyer study on 40 THA patients also used mediCAD^®^ software revealed the accuracy of 40%/ 77.5%/ 92.5% for cup size accuracy (± 0/ ± 1/ ± 2) and 37.5%/ 95%/ 100% for stem size accuracy (± 0/ ± 1/ ± 2) [37]. Also Schiffner et al. revealed accuracy (± 1) of 83.6% and 80.17% for stem and cup size, respectively [38]. In their study a [PGY] 4 resident (*n* < 500 THA) performed the 2D templating. Their results are more accurate than the current study. It shows that the software on its own can provide an acceptable facility to the assessor if used appropriately by an expert orthopedic surgeon.

When aiming for higher accuracy, 3D softwares show promising results [16, 26, 27]. However, it takes 10 times as much time as traditional 2D templating. The introduction of AI-based software has helped overcome this shortcoming [26, 27]. In the Huo et al. study using AI HIP software, 74.6% and 71.2% percent accuracy was achieved for the acetabular and femoral components, respectively, and less than 4 min was the average templating time. The accuracy was similar to the 3D mimics, but the templating time was significantly shortened, thereby making the operation more convenient. Also, it was more accurate than traditional 2D templating, with only a little bit longer templating time [26]. Ding et al. reported similar findings regarding the comparison between AI HIP and 2D templating [39].

The study’s retrospective nature is the main limitation of ours, cause not to control the bias that may happen. Another limitation is that we did not calculate accuracy if a more experienced person, such as an orthopedic professor, templates the implant size before the surgery and compares it to present results. An additional weakness of this study is that it did not take having spinal fusion surgery into account, and therefore did not consider how it might affect the quality of the radiographs, and hence the accuracy. However, severely impaired hip anatomy, as mentioned earlier, was not included and did not affect results. The large population of this study with multiple assessors during 4 years is a strength of this study.

## Conclusion

Our investigation showed that digital preoperative templating is somehow accurate and helpful in predicting cup and stem sizes in orthopedic patients using mediCAD^®^ by beginner orthopedic residents or fellow. Factors associated with increased stem size templating accuracy include female gender, DDH diagnosis, and Wagner Cone^®^. The smaller cup diameter and the residents performing the task are factors associated with increased cup size templating accuracy. Digital software like mediCAD^®^ remains favorable because of the short learning curve, user-friendly features, and low-cost maintenance, leading to level-up patient care and THA efficacy. Training the templating and instruction, using 3D templating, or getting help from AI are recommended to improve the accuracy. Further studies are necessary for clarifying the role of the assessor’s experience and expertise in THA preoperative templating.

## Data Availability

Not applicable.
